# Narratives boost entrepreneurial attitudes: Making an entrepreneurial career attractive?†

**DOI:** 10.1111/ejed.12274

**Published:** 2018-04-17

**Authors:** Katharina Fellnhofer

**Affiliations:** ^1^ LUT School of Business and Management, Lappeenranta University of Technology Lappeenranta 53851 Finland

## Abstract

This article analyses the impact of narratives on entrepreneurial attitudes and intentions. To this end, a quasi‐experiment was conducted to evaluate web‐based entrepreneurial narratives. The paired‐sample tests and regression analysis use a sample of 466 people from Austria, Finland, and Greece and indicate that individuals’ perceptions of the desirability of entrepreneurship and entrepreneurial intention are significantly different before and after exposure to entrepreneurial narratives. Furthermore, the findings indicate that perceptions of the feasibility of entrepreneurship are more strongly affected by videos than by cases. From a policy perspective, this study raises awareness that entrepreneurship is an attractive career path.

## 1 INTRODUCTION

Can entrepreneurial narratives inspire us to improve our entrepreneurial intentions? This question has not yet been conclusively answered, but several studies have emphasised that programmes in entrepreneurship education (EE) could have a positive impact on entrepreneurial intention (Peterman & Kennedy, 2003; Souitaris, Zerbinati, & Al‐Laham, 2007). This article analyses the potential of narratives in EE and answers the following question: Do multimedia narratives impact on entrepreneurial attitudes and intentions, and, if so, how?

In this context, theories regarding the phenomenon of narratives are theories of role recognition and social learning (Bandura, 1977; Gibson, 2004). Research has concluded that those who tell their stories could encourage others to choose a particular career path (Barling, Dupre, & Hepburn, 1998; Krumboltz, Mitchell, & Jones, 1976; Mitchell & Krumboltz, 1984). Hence, it seems that exposure to successful entrepreneurial narratives will affect an individual's intention and perceptions of the feasibility and desirability of an entrepreneurial career. According to Scherer, Adams, and Wiebe (1989), the observation of role models enables individuals to learn specific skills, knowledge and behaviours that are essential to embark on a new venture. In short, narratives can not only inspire readers and increase their motivation to choose a particular path, but also strengthen the traits and skills of aspiring entrepreneurs in the context of suitable formal or informal arrangements (Krumboltz et al., 1976). However, whether this is true for innovative pedagogic designs such as multimedia environments is still up for debate. This is particularly relevant for those who are (re)designing entrepreneurial courses to respond to researchers’ calls for more innovative pedagogic approaches to meet the needs of the multifaceted, creative and innovative world of entrepreneurship (Kuratko, 2005).

EE has received increasing attention globally and in related literature in recent decades (Bae, Qian, Miao, & Fiet, 2014; Kuratko, 2005; Lorz, Mueller, & Volery, 2013). However, despite the attention from the academic community, the multi‐contextual, multi‐disciplinary, multi‐functional, complex and diverse nature of EE raises many challenges regarding its development. Given the inherent interrelationship between EE and entrepreneurship, Davidsson and Wiklund (2001) stressed that studies should analyse the entrepreneurial phenomena under investigation at multiple supplementary levels. When reviewing past efforts which were based on traditional techniques, one can mention Kuratko's study which stressed that entrepreneurship educators should be cross‐disciplinary, innovative and entrepreneurially‐driven in order to convince readers to write about the entrepreneurial world from a pedagogical point of view: ‘As entrepreneurship educators, we must be the guardians of the true meaning and intent of the word ‘entrepreneurship*’* (Kuratko, 2005, p. 588).

Multimedia may play a large role in pedagogy. Therefore, web‐based entrepreneurial narratives may be particularly effective in changing readers’ attitudes and perceptions regarding an entrepreneurial career. As discussed in previous papers (Quimby & DeSantis, 2006), narratives can inspire their audience. Hence, this article presents the results of a quasi‐experiment using entrepreneurial narratives. The target group was an international audience of students attending a vocational school. We measured the effect of narratives on the participants’ entrepreneurial intention and their perceptions of the desirability and feasibility of such a career. The sample predominantly included students from Austria, Finland, and Greece. An immediate pre‐test–post‐test design was used for both the treatment and the control groups in order to allow for an unbiased difference‐in‐differences (DID) estimation of the effect of the treatment.

This article is divided as follows. First, to the best of our knowledge, it is the first study to evaluate the effect of entrepreneurial narratives. Studies that have a strong methodological character and work with control groups are crucial to further develop EE. Several recent important meta‐analyses on its effects have expressed the need for this type of studies (Bae et al., 2014; Martin, McNally, & Kay, 2013). Second, we assessed the immediate impact of attitudes on intention when assessing the impact of entrepreneurial narratives. Third, there is a lack of literature on the positive influence of EE (Lorz et al., 2013) and little agreement regarding pedagogical content (Davidsson, 2006; Mwasalwiba, 2010; Williams‐Middleton & Donnellon, 2014). Fourth, prior articles have stressed that higher education was crucial in supporting entrepreneurship (Crossouard, 2010; Greene & Saridakis, 2008; Hancock, 2011; Walsh, Hargreaves, Hillemann‐Delaney, & Li, 2015) which highlights that entrepreneurial narratives could represent a pedagogical instrument to facilitate its impact. Finally, the quasi‐experiment in this study identified the effect of initiatives in EE in an unbiased way.

To evaluate the impact of entrepreneurial narratives on EE, we selected validated items from related research. The results indicate that they have a significant negative impact on students’ desire to pursue an entrepreneurial career, whilst narratives presented in a multimedia format had a significant positive impact. The effects are stronger in the treatment group, but not significantly higher than in the control group. On average, the effect in the treatment group increased more than in the control group. The results are significant for entrepreneurial desirability and intention in the treatment group, but not in the control group. Therefore, entrepreneurial narratives are beneficial to students in higher education.

The results reveal the effect of the treatment. However, this effect may not be entirely related to the narratives. Because of the way in which the field experiment was set up, this study is unable to separately explore the effect of different mechanisms of the narratives. Our results are similar to those of other researchers investigating the same topic (BarNir, Watson, & Hutchins, 2011; Van Auken, Fry, & Stephens, 2006), supporting the idea that role models strongly influence entrepreneurial intention. The short‐term impacts of the findings of this study indicate that it may be more efficient to watch entrepreneurial stories than to carry out case studies. However, case studies may help students to gain a more nuanced and realistic understanding of what it means to be an entrepreneur. These positive effects may also be present over longer time periods.

## 2 THEORY

The role model theory served as the basis of this study. According to the construct of role models first presented by Gibson (2004), the entrepreneurial role model is ‘a cognitive construction based on the attributes of people in social roles that an individual perceives to be similar to him or herself to some extent and desires to increase perceived similarity by emulating those attributes’. In other words, Gibson stated that the expression ‘role model’ involved people's predisposition to interact and the concept of models which involves the psychological relation between one's own cognitive skills and behaviour patterns and those of another. Role models and their narratives tend to be perceived similarly in terms of personality, activities and objectives, which facilitates the learning of certain abilities or skills (Nauta & Kokaly, 2001). Role identification is a cognitive response that causes one's characteristics to match those of another individual (Kagan, 1958), especially one who is attractive (Bell, 1970). It can also cause an individual's interests to be altered or adjusted (Witt & Wilson, 1991) to imitate the role model (Kagan, 1958). Rahman and Day (2014) suggest that this role model should be an empathetic individual who expresses him‐ or herself via entrepreneurial narratives. Accordingly, a role model can strengthen the traits and skills of aspiring entrepreneurs via formal or informal arrangements.

There are other theories regarding role models or narratives that are related to theories of role recognition and social learning (Bandura, 1977; Gibson, 2004). Narratives can inspire and motivate individuals to choose a particular path (Krumboltz et al., 1976). An individual selects a role model through recognition based on his or her reputation and charisma as an entrepreneur. Hitherto, the discourse regarding the link between career choice and role models’ narratives has taken two central paths (Quimby & DeSantis, 2006). On the one hand, the social cognitive career theory (Lent, Brown, & Hackett, 1994) states that narratives provide support and thus have a direct effect on one's career choice. On the other, studies have demonstrated that role models strongly influenced one's entrepreneurial intention and activities (BarNir et al., 2011; Van Auken et al., 2006). Much research has found that successful entrepreneurial role models had a positive impact on individuals’ attitudes towards entrepreneurship and perceived behaviour control (BarNir et al., 2011; Boyd & Vozikis, 1994; Krueger, 1993; Nauta & Kokaly, 2001; Scherer, Brodzinski, & Wiebe, 1991). Based on these findings, we expect a similarly strong link between attitudes and entrepreneurial narratives. Overall, the theoretical and empirical evidence demonstrate that entrepreneurial role models play a crucial role in generating entrepreneurial interest (Wilson, Marlino, & Kickul, 2004). However, no studies have investigated the effect of entrepreneurial narratives via multimedia platforms. In particular, multimedia represents a pedagogical avenue which has a high potential. Digital videos offer a promising alternative to traditional techniques (Rogers & Coughlan, 2013). We based our work on the *Theory of Multimedia Learning* (Mayer, 2001, 2005) and the *Cognitive Load Theory* (Paas, Renkl, & Sweller, 2003; Paas & Sweller, 2014; Sweller, 1988, 1999; Valcke, 2002).

Mayer's theory (2001, 2005) proposes that, first, visual and auditory information are managed via different channels. This is based on the ideas of the *Dual Coding Theory* (Clark & Paivio, 1991; Paivio, 1991; Paivio & Csapo, 1973) which stress that human working memory involves two independently activated but interconnected channels to deal with information (Rieber, 1994). The second assumption is that the capacity of each channel is limited. This is in line with the *Cognitive Load Theory* which also stresses that our memory and attention are limited. This limitation relates to an intrinsic process which describes a student's focus on the learning content. In addition, this theory postulates cognitive representations and its incorporation with previous learned representations. Finally, this is also in line with Mayer's theory (2001, 2005) that understands learning as an active procedure by building mental representations of knowledge and adding them to previously acquired knowledge.

A substantial amount of literature has described the intention to found a company as a phase in the process of starting a business (Kolvereid, 1996; Krueger, Reilly, & Carsrud, 2000; Tegtmeier, 2006). The most frequently cited theory here is that of planned behaviour which measures the degree of intention as a direct antecedent of behaviour (Ajzen, 1991, 2011). One study indicates that the relationship between intention and behaviour exists regardless of age, gender, experience, education, and entrepreneurial ambition (Kautonen, Gelderen, & Fink, 2013). It builds on the theory of planned behaviour and explores the impact of innovative pedagogical efforts on entrepreneurial intention with a particular focus on multimedia‐based entrepreneurial narratives.

## 3 HYPOTHESES

Previous research on EE has not adequately discussed the effects of entrepreneurial stories that are disseminated via multimedia. Only a few researchers have studied approaches to narratives (Denise & Watson, 2007; Godsey & Sebora, 2009; Harmeling & Sarasvathy, 2013). More than ten years ago, in Kuratko's (2003, 2005) studies on the development, trends and challenges of EE, the importance of innovation‐driven approaches and designs in EE was stressed as an international economic force. Kuratko emphasised that progress in this area depended on a continuous expansion of the range of tactics and pedagogies applied in EE. In line with these recommendations and those of other scholars investigating EE (Lautenschläger & Haase, 2011; Solomon, Duffy, & Tarabishy, 2002) and the European Commission (2013), this study assesses the impact of a multimedia platform that promotes entrepreneurial narratives on EE in which successful entrepreneurs tell their stories via multimedia, offering insights into entrepreneurship.

According to Pfeffer's (1982) fundamental role theory, role models are not required to satisfy role expectations. Previous studies stress that portraiture provides a fuller, more nuanced and more truthful account of identity (Rippin, 2012). In other words, a sense of theatre seems vital for business, pedagogy and research (Spence, 2007). Gliner, Goldman and Hubert (1983) note that an introduction to narratives influences how individuals portray information. The philosophical understanding of the relationship between narratives and the power of the narrative approach in management and enterprise research is improving (Hamilton, 2006). Prior research introduced a framework that explained the roles of narratives and emotion in entrepreneurship communication in increasing students’ understanding (Godsey & Sebora, 2009; Roundy, 2014). Colleagues defined networking support as the assumption that telling others’ narratives supported business (Bisht, 2013). Other recent research presents narratives that explore and share individuals’ personal experiences with establishing businesses (Karmali, 2012; Wadhwani & Chen, 2011). Prior storytelling research on the entrepreneurial identities of Bill Gates and Richard Branson applied narratives and used the Internet to invite viewers to watch these (Boje & Smith, 2010). Self‐defining narratives provide comprehensive views of individuals from the perspective of personality psychology (McAdams & Pals, 2006).

From a cross‐cultural perspective, optimism in narratives promotes the resilience that is crucial to maintain entrepreneurship (Junaid, Durrani, Mehboob ur, & Shaheen, 2014). Kant's concept of maturity is often applied to discuss entrepreneurs’ narrative articulation of objectives related to public, social and moral concerns (Clarke & Holt, 2010). Narratives are a valuable tool to learn entrepreneurship management (Friedman & Prusak, 2008; Gabriel & Connell, 2010) and can contribute to its theoretical understanding. Furthermore, they are a novel way to learn through emergent and relational processes that allow for the realisation of entrepreneurial opportunities (Denise & Watson, 2007). Researchers have stressed the importance of using the narrative approach as an entrepreneur (O'Connor, 2002).

Using a holistic narrative approach, a career counseling framework and method of practice Zikic and Franklin (2010) seek to discover and value lives in narratives. Hood and Young (1993) believe that most aspects of entrepreneurship can be learned via narratives (Boje & Smith, 2010; Denise & Watson, 2007; Down & Warren, 2008; Johansson, 2004; Linstead & Hytti, 2005; Matlay & Harmeling, 2011; Rae, 2005). Because storytelling involves the sharing of experiences and knowledge, it seems valuable and effective in EE to facilitate the creation of entrepreneurial identities (Donnellon, Ollila, & Middleton, 2014).

*H1: Multimedia entrepreneurial narratives have a positive effect on the overall perception of entrepreneurial role models*.

Using a pre‐test–post‐test control group research design, Peterman and Kennedy (2003) found that EE participation had a significantly positive effect on the perceived desirability and feasibility of entrepreneurship among secondary school students. Later, Chen, Su, and Wu (2012), using a sample of 230 nascent American entrepreneurs, found that ambitious entrepreneurs who had received EE tended to take more risks. In line with other research

(Byabashaija & Katono, 2011; Kuehn, 2008), Wurthmann's (2014), multiple mediation analysis found that the perceived desirability and feasibility of entrepreneurship were associated with the creation of a business. Grünhagen and Berg (2011) found that regulatory reliability, complexity and policy supportiveness increased the perceived feasibility and desirability of growth, stressing the importance of perceptions regarding desirability and feasibility. Whilst past research in other disciplines has implied that there was a direct effect between perceived desirability and feasibility, Douglas and Fitzsimmons’ (2013) international study found negative relations in the framework of entrepreneurial intentions, suggesting the presence of a new type of entrepreneur. Thus, we propose the following hypotheses with the expectation that the effects of multimedia entrepreneurial stories are stronger than traditional narratives and therefore that the new pedagogical approach is effective and useful:

*H2: Multimedia entrepreneurial narratives have a positive impact on the perceived desirability of entrepreneurship*.

*H3: Multimedia entrepreneurial narratives have a positive impact on the perceived feasibility of entrepreneurship*.

Shepherd and Krueger (2002) identify the antecedents of entrepreneurial thinking using an intention‐based model of the social cognition of entrepreneurial teams, concluding that individuals’ perceptions of desirability and feasibility play a primary role in the planning required to start a business. Furthermore, Diaz‐Garcia, Saez‐Martinez, and Jimenez‐Moreno (2015) found that the entrepreneurial intentions of students receiving EE were greater than those of participants in the control group and increased over time. The exploration of opportunities differs significantly between individuals, suggesting educational potential for pedagogic instruments for future entrepreneurs (Gilad, Kaish, & Ronen, 1988; Ucbasaran, 2004). In this framework, familiarity with inspecting relevant information channels is important (Fiet, 2007; Raluca, 2013; Robinson, 2014). Previous scholars have noted the importance of experience for the exploration of opportunities (Shane & Venkataraman, 2000) and that this experience could be transferred to potential entrepreneurs through narratives. Shane and Venkataraman (2000) highlight two things that impact entrepreneurial discovery: 1 ‘… the possession of prior information necessary to identify an opportunity and 2 the cognitive properties necessary to value it’ (p. 221f). Entrepreneurial narratives can provide readers with experience because there are many channels through which they can be transmitted (e.g., Internet). This increases the probability that readers will analyse a potential business opportunity.

According to Ardichvili, Cardozo, and Ray (2003), entrepreneurial awareness, information asymmetry, previous understandings, networks, personality traits, confidence, self‐efficacy, creativity and the various types of opportunities are what lead to the recognition of potentially successful opportunities.

Entrepreneurial self‐efficacy can improve essential skills that are required throughout the business development process. One's perceived self‐efficacy is often more of a determining factor for future actions than concrete skills and knowledge (Krueger & Dickson, 1994). The concept of self‐efficacy refers to an individual's decision and determination to act, reflection practices and emotional responses to barriers (Bandura, 1997; Lent et al., 1994). In addition, it has been linked to taking risks and exploring entrepreneurial opportunities (Krueger & Dickson, 1994) as well as career choice (Bandura, 1986) and work performance (Chen, Casper, & Cortina, 2001). Scholars have recommended that policymakers and educators improve individuals’ entrepreneurial intentions by increasing their self‐confidence and improving their expectations regarding an entrepreneurial career (Segal, Borgia, & Schoenfeld, 2002).

Entrepreneurial intentionality has been identified as an indicator of the value of EE (Bae et al., 2014; Fayolle & Gailly, 2013). It is discussed in other disciplines, such as social psychology, as planned behaviours (Krueger et al., 2000). Recently, it has gained attention in the field of entrepreneurship research (Kolvereid, 1996; Krueger et al., 2000). Inventors believe that offering EE boosts entrepreneurial intentions. However, few scholars anticipated a positive association between EE and entrepreneurial intention (Sánchez, 2013).

In line with prior work, this study emphasises that perceptions and attitudes predict behavioural intentions (Kolvereid, 1996). To increase the amount of research on the relationship between entrepreneurial intention and education, since intention is not the same as behaviour (Armitage & Conner, 2001), this empirical research investigates perceived behaviour control and behaviour linked to already‐performed actions. Thus, we propose the following hypothesis, assuming that, due to the effectiveness of the pedagogical tool, the effects of the treatment will be even stronger after the experiment:

*H4: Multimedia entrepreneurial narratives have a positive impact on perceived entrepreneurial intention*.

## 4 METHODS

### 4.1 Research design and sample

In this study, we used a quasi‐experimental research design where participants responded to a survey on their entrepreneurial role models, entrepreneurial intentions and perceptions of the desirability and feasibility of an entrepreneurial career. Respondents were then exposed to an online entrepreneurial video narrative and asked to take the survey again. We applied seven multimedia entrepreneurial narratives to the treatment group and used videos of real company cases for the control group. This research design has several drawbacks. First, it is unable to measure long‐term treatment effects. However, we were able to measure the frequency of short‐term effects. Additionally, according to the literature, the short‐term effects of narratives on entrepreneurial intentions may also affect individuals for longer periods (Cunha & Heckman, 2007). Moreover, the setting of the quasi‐experiment enabled the researcher to investigate the overall treatment effect of entrepreneurial narrative videos.

The data were collected from February 2016 to May 2017 from participants who were mainly from Austria, Greece, and Finland. In total, the treatment group consisted of 426 observations and 40 were placed in the control group. From these 466 observations, 123 (26.4%) stressed that they had participated in entrepreneurship education. Most (343 observations, 73.6%) had not been engaged in entrepreneurship education before. They filled out questionnaires before and after the treatment or control which took place during regular curriculum. Overall, the data were collected over a long period of time in different countries at different times. We wanted to test the multimedia narratives and the pre‐post questionnaire in Finland first and we then developed the material for the control group and collected data. The students were not randomly assigned to the groups but self‐selected from the website. We admit that this is a weakness of our research design, but we carried out a number of analyses to verify that the difference in sample sizes, nationalities and fields of study in the two groups did not bias our results. For instance, the groups are similar in terms of gender, age and prior exposure to entrepreneurial role models (Tables 1 and 3). Second, in Table 4 we find the second regression model that controls for the effects of nationality and field of study. The parameter estimates are unaltered. Third, we conducted a robustness check analysis by randomly selecting the same number of observations (n = 40) from the treatment group as from the control group. The results indicated no significant new results compared to those of the whole sample.

**Table 1:** Differences in the control variables

	% of Total			Pearson Chi‐Square Test
Control variable	*Control (C) n = 40*	*Treatment (T) n = 426*	*C + T*	*Asymp. Sig. (2‐sided)*
Gender				
Female (n = 180)	52.5	37.3	38.6	
Male (n = 286)	47.5	62.7	61.4	0.059*
*Total*	*100*	*100*	*100*	
Age				
<18 (n = 31)	0.0	7.3	6.7	0.36 (n.s.)
18–24 (n = 348)	87.5	73.5	74.7	
25–34 (n = 55)	10.0	12.0	11.8	
35–44 (n = 19)	2.5	4.2	4.1	
45–55 (n = 11)	0.0	2.6	2.4	
56< (n = 2)	0.0	0.5	0.4	
*Total*	*100*	*100*	*100*	
Nationality				0.001***
Austria (n = 172)	30.0	37.6	36.9	
Greece (n = 103)	70.0	30.0	33.5	
Finland (n = 156)	0.0	24.2	22.1	
Other (n = 35)	0.0	8.0	8.0	
*Total*	*100*	*100*	*100*	
Field of Study				0.000***
Business & Management (n = 190)	70.0	38.0	40.8	
Information Technology (n = 22)	0.0	5.2	4.7	
Natural Sciences (n = 21)	0.0	4.9	4.5	
Technical Sciences (n = 129)	0.0	30.3	27.7	
Social Sciences (n = 46)	0.0	10.8	9.9	
Arts (n = 29)	25.0	4.5	6.2	
Other Sciences (n = 29)	5.0	6.3	6.2	
*Total*	*100*	*100*	*100*	

*Notes*. * indicates significance at the 10% level. *** indicates significance at the 1% level.

### 4.2 Entrepreneurial narratives in multimedia format

Before discussing the outcome variables, it is important to understand which kinds of narratives contributed to the overall effect of the treatment. Several tests, although imperfect, were carried out. Before the tests, we took care to choose appropriate entrepreneurial narratives. We gathered those that covered different sectors (e.g., ICT, energy, transportation, consulting, event management, real estate, finance and merchants), age groups (from 30 to 65 years of age), sex (females and males), company size (one large company, five small‐ and medium‐sized companies and one sole proprietor), different countries (Finland, Austria, Germany and Spain) and different languages (German, Finnish, English and Spanish). A structured guide led the interviews, so all the web‐based stories have a similar structure despite wide variations in content. The senior entrepreneurs narrowed their success and failure stories and how they assessed entrepreneurial opportunities. Additionally, they were asked to provide recommendations for nascent entrepreneurs. During their stories, important key recommendations or story elements were highlighted as subtitles. Overall, the videos lasted between four and ten minutes.

### 4.3 Measurements

The survey measures were based on validated topics from EE literature (De Clercq, Honig, & Martin, 2013; Kolvereid, 1996; Krueger et al., 2000; Kuehn, 2008; Liñán & Chen, 2009; Peterman & Kennedy, 2003; Wurthmann, 2014). How participants were inspired by a role model was measured using a modified 7‐point Likert scale developed by Nauta and Kokaly (2001) which included the following statements: ‘There is an entrepreneurial person I am trying to be like in my career pursuits’; ‘There is an entrepreneurial person particularly inspirational to me in my career path’; ‘In the career path I am pursuing, there is an entrepreneurial person I admire’; ‘I have a mentor in my potential entrepreneurial career field’; and ‘I know of an entrepreneurial person who has a career I would like to pursue’. Mean, standard deviation (SD) and item total correlation Alpha if item is deleted are provided for each item in Appendix table A1‐A8 for the pre‐assessment data and B1‐B8 for the post‐assessment data.

#### 4.3.1 Control variables

We controlled for individual characteristics, including age, gender, field of study and nationality. Table 1 illustrates the differences between the treatment and control group in the control variables. Because growing evidence, particularly the findings of Dodd and Hynes’ (2012) six‐country study, has exposed differences in entrepreneurial narratives due to regional differences, we controlled for nationality. Ever since research has shown that females tended to have less entrepreneurial intentions than males (Hao, Seibert, & Hills, 2005) up to date gender issues regarding EE were discussed as effects that were still inadequately understood (Chowdhury, 2005; Fellnhofer, Puumalainen, & Sjögrén, 2016). Table 2 stresses the gender differences regarding entrepreneurial desirability (p‐value_t=0_ = 0.054* and p‐value_t=1_ = 0.021**), entrepreneurial feasibility (p‐value_t=0_ = 0.00*** and p‐value_t=1_ = 0.00***) and entrepreneurial intention (p‐value_t=0_ = 0.012** and p‐value_t=1_ = 0.003***) in the treatment group.

**Table 2:** Gender differences

			*Control (C) n = 40*						*Treatment (T) n = 426*					
			N	Mean	SD	SE	Sig. (2‐tailed)	Mean Difference	N	Mean	SD	SE	Sig. (2‐tailed)	Mean Difference
Inspiration/Modeling	Pre	Female	21	3.75	1.59	0.35	0.26	−0.47	159	4.06	1.22	0.10	.624	0.06
		Male	19	4.22	0.83	0.19			267	3.99	1.37	0.08		
	Post	Female	21	3.88	1.54	0.34	0.63	−0.21	159	3.94	1.30	0.10	.228	−0.16
		Male	19	4.08	1.07	0.25			267	4.10	1.35	0.08		
Desirability	Pre	Female	21	3.67	1.64	0.36	0.13	−0.72	159	4.29	1.55	0.12	0.054*	−0.29
		Male	19	4.39	1.23	0.28			267	4.58	1.44	0.09		
	Post	Female	21	3.54	1.55	0.34	0.13	−0.69	159	3.98	1.69	0.13	0.021**	−0.38
		Male	19	4.23	1.25	0.29			267	4.36	1.62	0.10		
Feasibility	Pre	Female	21	3.13	1.11	0.24	0.068*	−0.59	159	3.33	1.03	0.08	0.00***	−0.53
		Male	19	3.73	0.86	0.20			267	3.86	1.19	0.07		
	Post	Female	21	3.13	1.03	0.23	0.31	−0.33	159	3.44	1.15	0.09	0.00***	−0.47
		Male	19	3.46	0.98	0.23			267	3.91	1.36	0.08		
Intention	Pre	Female	21	3.44	1.35	0.30	0.58	−0.21	159	3.52	1.56	0.12	0.012**	−0.38
		Male	19	3.65	1.02	0.23			267	3.90	1.49	0.09		
	post	Female	21	3.63	1.51	0.33	0.67	−0.19	159	3.57	1.55	0.12	0.003***	−0.46
		Male	19	3.82	1.23	0.28			267	4.03	1.54	0.09		

*Notes*. * indicates significance at the 10% level. ** indicates significance at the 5% level. *** indicates significance at the 1% level.

Table 3 illustrates how the participants differ in terms of important entrepreneurship criteria such as experience with role models. They are ranked on a scale from 1 (strongly disagree) to 7 (strongly agree) if they have an entrepreneurial role model and who it is. Overall, there is no significant difference in means between the treatment and the control group when individuals assess potential role models such as parents or siblings, friends or someone who is important to her/him and/or someone she/he does not know personally.

**Table 3:** Experience with entrepreneurial role models

	*Control (C) n = 40*		*Treatment (T) n = 426*		*C + T n = 466*		t‐test for Equality of Means Equal *variances assumed*	
If you have an entrepreneurial role model, who is it? Participants evaluated the statements from 1 (strongly disagree) to 7 (strongly agree).	Mean	SD	Mean	SD	Mean	SD	Sig. (2‐tailed)	Mean Difference
Parents or siblings	3.60	1.766	3.94	1.928	3.91	1.916	0.285	−0.339
Friends	3.58	1.599	3.96	1.706	3.92	1.699	0.173	−0.383
Someone else who is important to me and/or someone I do not know personally	4.00	1.664	4.34	1.691	4.31	1.690	0.224	−0.340

## 5 RESULTS

To study the power of multimedia presentation of entrepreneurial narratives, a difference‐in‐differences (DID) analysis and repeated ANOVA measures which employed the general linear model (GLM) procedure in SPSS software were performed. The values of the variables under investigation for an individual in the treatment group (T) before the start of the entrepreneurial narrative (t = 0) were designated as μ_T,0,_ whilst those after the treatment period (t = 1) were presented as μ_T,1_. This notation is the same for the control group (C). Before the traditional entrepreneurial case study starts at t = 0, we measured μ_C,0._ After the case study, we measured μ_C,1_ at t = 1. The average variations per variable between the pre‐test and post‐test for all individuals in the treatment and control groups are indicated by Δμ_T_ and Δμ_C_, respectively. Hence, the results of the DID are calculated using the following equation:

(1) $$\delta \;=\;\Delta \mu_{T}-\;\Delta \mu_{C}$$

Table 4 illustrates the differences in means between the treatment and control groups before watching the video or performing the case study at t = 0 and at t = 1. While the mean inspiration in the treatment group at t = 1 is slightly higher than in the control group (μ_T,1_ = 4.04 and μ_C,1_ = 3.98), the mean entrepreneurial intention in the treatment group increased less than in the control group at t = 1 (μ_T,1_ = 3.86 and μ_C,1_ = 3.73). Table 4 also outlines differences in the descriptive statistics of outcome variables for both groups, which were calculated using the equation Δμ = μ_1_ − μ_0_. Furthermore, Table 4 illustrates the results of paired samples tests performed for the groups separately and together. There is a significant difference between the mean entrepreneurial desirability (Δμ_T_ = −0.25, t = −4.78***) in the treatment group and that in the overall sample (Δμ_T_ = 0.25, t = −4.96***), but not in the control group (Δμ_C_ = −0.14, t = −1.50, n.s.). In addition, the paired samples test revealed a significant difference in the feasibility of an entrepreneurial career at the 10% level in the treatment group (Δμ_T_ = 0.07, t = 1.69*), but not in the control group (Δμ_C_ = −0.13, t = −1.16, n.s.) or the whole group (Δμ_TC_ = 0.05, t = 1.37, n.s.). Finally, a significant difference between the mean entrepreneurial intention (Δμ_T_ = 0.10, t = 2.56***) in the treatment group and in the whole sample (Δμ_T_ = 0.11, t = 2.90***) was detected, but not in the control group (Δμ_C_ = 0.19, t = 1.64, n.s.).

**Table 4:** Descriptive statistics and paired samples test

	t = 0		t = 1		ΔμT				
	Mean	SD	Mean	SD	Mean	SD	SE	t	p‐value
*Treatment and control group*									
Inspiration/Modeling	4.01	1.31	4.04	1.33	0.02	1.10	0.05	0.45	0.65
Entrepreneurial Desirability	4.43	1.49	4.19	1.64	−0.25	1.07	0.05	−4.96	0.00
Entrepreneurial Feasibility	3.64	1.15	3.70	1.28	0.05	0.86	0.04	1.37	0.17
Entrepreneurial Intention	3.74	1.50	3.85	1.54	0.11	0.81	0.04	2.90	0.00
*Control group*									
Inspiration/Modeling	3.98	1.29	3.98	1.33	0.00	0.89	0.14	0.00	1.00
Entrepreneurial Desirability	4.01	1.49	3.87	1.44	−0.14	0.60	0.09	−1.50	0.14
Entrepreneurial Feasibility	3.42	1.03	3.29	1.01	−0.13	0.68	0.11	−1.16	0.25
Entrepreneurial Intention	3.54	1.20	3.73	1.37	0.19	0.72	0.11	1.64	0.11
*Treatment group*									
Inspiration/Modeling	4.02	1.31	4.04	1.33	0.02	1.12	0.05	0.47	0.64
Entrepreneurial Desirability	4.47	1.49	4.22	1.65	−0.25	1.11	0.05	−4.78	0.00
Entrepreneurial Feasibility	3.66	1.16	3.73	1.30	0.07	0.87	0.04	1.69	0.09
Entrepreneurial Intention	3.76	1.53	3.86	1.56	0.10	0.81	0.04	2.56	0.01

*Notes*. * indicates significance at the 10% level. ** indicates significance at the 5% level. *** indicates significance at the 1% level.

In our methodological approach, we relied on prior studies (Huber, Sloof, & Van Praag, 2014; Rosendahl Huber, 2015). Twofold differencing eliminated possible biases that were unrelated to the narratives (Imbens & Wooldridge, 2009). This is not a critical issue because this investigation only assesses the immediate short‐term effects of the treatment. For each individual (i = 1, … . N), the following variables are observed: D_i_; μ_i,0_; μ_i,1_; X_i,0_; and X_i,1_. D_i_ is a dummy variable that takes a value of 1 if individual i was part of the treatment group, μ_i,t_ is the value under investigation for individual i at time t, and X_i,t_ is a vector of the control variables for individual i at time t. The difference, Δμ = μ_i,1_ ‐ μ_i,0_, is then regressed on the treatment indicator, D_i_, and the lagged outcome, μ_i,0_:

(2) $$\Delta \mu_{i}=\;\alpha \;+\;\delta D_{i}+\;\beta \mu_{i,0}+\;\epsilon_{i}$$

To explain the results of the different variables under investigation, standardised and explanatory variables were applied. Additionally, the baseline value for the variable under investigation was used to determine the latent ceiling effect. So, if the initial value is rather high, there is less room for increases after the treatment. To check the robustness of the coefficients estimated by Equation (2), a vector of control variables (X_i_), including age, gender, field of study and nationality was embedded. A simple level regression yields approximate results, like the DID. The approximate results of calculating Equation (2) using the value of the outcome variable at t = 1 (μ_i,1_) as the dependent variable both with and without control variables are presented in the next section. The development of the outcome variables is more interesting than the level of these parameters at a certain point in time. The results of the DID estimation of Equation (2) are shown in Table 5 which illustrates the unstandardised coefficients for both groups and the net treatment effect, δ. In columns 3 and 4, F and R^2^ refer to regressions without control variables and columns 7 and 8 list regressions that take the control variables into account. A significant difference in the perceived feasibility of an entrepreneurial career (B = 0.077*) is detected, leading to the conclusion that videos significantly affect the perceived feasibility of entrepreneurship, but not its desirability or individuals’ entrepreneurial intention.

**Table 5:** Treatment effects

	Without control variables				With control variables				DID
	SE	B	F	R^2^	SE	B	F	R^2^	δ
Inspiration/Modeling	0.167	0.10 (n.s.)	45.212	0.163***	0.181	−0.049 (n.s.)	4.735	0.205***	0.03
Entrepreneurial Desirability	0.174	−0.011 (n.s.)	11.184	0.046***	0.19	−0.051 (n.s.)	1.625	0.081**	−0.1
Entrepreneurial Feasibility	0.139	0.077*	11.662	0.048***	0.148	0.072 (n.s.)	2.638	0.126***	0.2
Entrepreneurial Intention	0.131	−0.021 (n.s.)	11.618	0.048***	0.141	−0.035 (n.s.)	2.072	0.101***	0.01

*Note*. Standardised Coefficient B is presented. The estimates in each cell come from separate regressions. All regressions control for the base line level of the outcome variable. * indicates significance at the 10% ‐level. ** indicates significance at the 5% ‐level. *** indicates significance at the 1%‐level.

## 6 DISCUSSION AND CONCLUSION

Considering the importance of innovative EE, we assessed the short‐term impact of entrepreneurial narratives on the perceived desirability and feasibility of entrepreneurship as well as entrepreneurial intention of individuals of different ages, nationalities and educational levels. The narratives were presented by individuals from different countries (Finland, Austria, Germany and Spain) who were active in different sectors, such as ICT, energy, transportation, consulting, event management, real estate, the financial sector and the merchant sector and were employed by companies ranging from one large company to five small‐ and medium‐sized companies, to one sole proprietor. By conducting a quasi‐experiment from February 2016 to May 2017 primarily in Finland, Austria, and Greece, we achieved estimates for the sample. Overall, our findings stress that entrepreneurial narratives have a significant impact on the development of entrepreneurial perceptions. However, the effect is both negative and positive. While the innovative pedagogical tool allows one to strengthen one's perception of the feasibility of an entrepreneurial career, its desirability is negatively influenced (but not significantly). Thus, multimedia entrepreneurial narratives have a significant effect on the perceived feasibility of entrepreneurship. The results for all outcome variables were stronger in the treatment group than in the control group.

### 6.1 Theoretical and practical implications

Since EE has received growing attention worldwide and many university‐based entrepreneurship ecosystems are developing (Hancock, 2011; Rees, 2010), evaluating the effectiveness of entrepreneurial narratives is a first essential step to virtual access to a worldwide network of entrepreneurial narratives. As a promising field of research, EE will continue to arouse the interest of a wide range of individuals, including researchers, lecturers, policy makers, (nascent) entrepreneurs and society at large. For this reason, we not only discuss the theoretical implications for EE researchers but also mention the practical implications of the research for policy makers, (nascent) entrepreneurs and society at large.

Our results suggest that multimedia entrepreneurial narratives seem to be an effective tool for promoting entrepreneurship across the globe. With respect to the key role of entrepreneurs in economic development, the assessment of tools to stimulate entrepreneurial attitudes, perceptions and intentions is of great value to both academics and practitioners. Hence, our results recommend that innovation‐driven EE involving web‐based entrepreneurial narratives appears to be an effective and appropriate way to positively change individuals’ perception of entrepreneurship. It is not a universal solution, but the significant short‐term effects of the treatment should be considered in EE policy involving guided limitations.

Considering the previously discussed topics that seemed to be under‐researched (Lorz et al., 2013), web‐based narratives of different entrepreneurs that use re‐narrating practices to discuss the field of entrepreneurship may enhance previous studies. In this way, the theoretical and practical implications of this study will be instrumental to the field of EE and society as a whole. As recommended by the European Commission (2013), entrepreneurs are not recognised or rewarded enough to act as role models and stimulate the growth of the European economy. In addition to playing roles in designing programmes for EE and creating appropriate business environments, entrepreneurial narratives can inspire potential entrepreneurs. Although much research on EE has been published in recent decades, little has called for action. The findings of this study highlight that the innovative aspects of EE should receive more attention from scholars and the creators of EE programmes, EE institutions and centres and policy makers. This study is the first to evaluate the effects of multimedia entrepreneurial narratives on individuals. We assessed their immediate effects, showing that they have a significant influence on certain perceptions, which in turn affect entrepreneurial intention. However, it was also found that some perceptions changed significantly negatively. Overall, the research findings are not only interesting for the academic community, but also for (re)designing EE courses to increase their effectiveness. However, there is still little research on such innovation‐driven educational approaches (Kuratko, 2005) and further efforts are required to reveal different treatment effects in more detail.

### 6.2 Limitation

Like any other academic effort, this research and its approaches have inherent shortcomings, and despite our robustness checks, readers should be aware of the limitations of the applied methods. First, the study could be criticized for its broad range, which may lead to bias in the discussion of the current body of knowledge. Second, the study only examined the short‐term impact of seven entrepreneurial narratives and the design does not allow assessment of the longer‐term effects of an innovative approach to EE. However, revealing the short‐term effects is a step towards a better perception of the effects of innovative approaches in EE, such as multimedia entrepreneurial videos and the validity of dynamic spillover effects in the field of EE. Third, the evidence shows that, unfortunately, the current set‐up of this field experiment prevents us from unscrambling the effects which future studies could focus on. Fourth, the control group was made up of a small sample. Fifth, there is diversity in the entrepreneurial narratives and sample. As the first study investigating innovative approaches to EE, this structure seems to be adequate. Nevertheless, it would be worth knowing whether and to what extent a more representative sample from different countries, including unemployed individuals, would impact the results. It may be possible that other types of entrepreneurial narratives have different impacts. Although the limitations of our contribution are not significant, our discussion should be read carefully. To overcome these limitations, future research should examine the nature and impact of multimedia entrepreneurial stories in relation to a wider population and geographical scope, perhaps with a longitudinal design and applied measures.

Compared to the treatment group (n = 426), the control group (n = 40) is rather small. Thus, we carried out an additional analysis such as weight and selected cases to compare the results if the treatment group would also consist of 40 cases (randomly selected cases out of the treatment group) and the results if the control group would also consist of approximately 400 cases (10% weight cases of the control group). Overall, the results of these additional analyses show no significant different effects than the prior results. However, this difference in group size represents a weakness which needs to be considered. There was no particular reason to work with only 40 in the control group. While during the quasi‐experiments individuals were asked to choose an entrepreneurial video, the data of the control group were gathered via individuals who were free to choose the treatment or control group online at a later stage. The research platform is available online. Nevertheless, this research design offers room for improvement. The control group could be increased so that the samples are equal.

Despite its limitations, this study is a valuable contribution to EE research. The integrity of the study is ensured by a diverse set of reliable sources, applied tools and quality checks. It is the first evaluation of multimedia entrepreneurial narratives in EE and highlights the need to enrich and enlarge the field of EE literature as a whole.

## APPENDIX 1

Mean, standard deviation (SD) and item total correlation Alpha if item is deleted are provided for each item in Appendix table A1, A2, A3, A4, A5, A6, A7, A8 for the pre‐assessment data and B1, B2, B3, B4, B5, B6, B7, B8 for the post‐assessment data.

**Table A1:** *Pre‐assessment*: Entrepreneurial role model—Inspiration/Modeling (modified from Nauta & Kokaly, 2001)

Domain	Item	Mean	SD	Item total correlation	Alpha if item is deleted
IM_01	There is an entrepreneurial person I am trying to be like in my career pursuits	4.14	1.62	.725	.821
IM_02	There is an entrepreneurial person particularly inspirational to me in my career path	4.16	1.62	.772	.809
IM_03	In the career path I am pursuing, there is an entrepreneurial person I admire	4.26	1.59	.725	.822
IM_04	I have a mentor in my potential entrepreneurial career field	3.36	1.65	.542	.867
IM_05	I know of an entrepreneurial person who has a career I would like to pursue	4.15	1.68	.647	.841

*Note*. Participants evaluated the statements from 1 (strongly disagree) to 7 (strongly agree).

**Table A2:** *Pre‐assessment*: Perceived entrepreneurial desirability (Modified from Peterman & Kennedy, 2003)

Domain	Item	Mean	SD	Item total correlation	Alpha if item is deleted
D_1	I would love to start my own business	4.39	1.75	.814	.799
D_2	I would be very tense to start my own business	4.40	1.55	.701	.896
D_3	I would be very enthusiastic to start my own business	4.50	1.66	.814	.798

*Note*. Participants evaluated the statements from 1 (strongly disagree) to 7 (strongly agree).

**Table A3:** *Pre‐assessment*: Perceived entrepreneurial feasibility (Modified from Peterman & Kennedy, 2003)

Domain	Item	Mean	SD	Item total correlation	Alpha if item is deleted
F_1	It will be easy to start my own business	2.81	1.53	.624	.723
F_2	I will be successful when I have my own business	4.31	1.40	.551	.748
F_3	I won't be overworked when I have my own business	3.29	1.60	.541	.750
F_4	I know enough how to start a business	3.08	1.68	.644	.714
F_5	I am sure about myself	4.71	1.62	.452	.780

*Note*. Participants evaluated the statements from 1 (strongly disagree) to 7 (strongly agree).

**Table A4:** *Pre‐assessment*: Perceived entrepreneurial intention (Modified from Liñán & Chen, 2009)

Domain	Item	Mean	SD	Item total correlation	Alpha if item is deleted
I_1	I am ready to do anything to be an entrepreneur	3.28	1.65	.770	.941
I_2	My professional goal is to become an entrepreneur	3.66	1.71	.831	.934
I_3	I will make every effort to start and run my own firm	3.64	1.63	.869	.930
I_4	I am determined to create a firm in the future	3.92	1.69	.864	.930
I_5	I have very seriously thought of starting a firm	3.89	1.77	.830	.934
I_6	I have the firm intention to start a firm some day	4.05	1.72	.824	.935

*Note*. Participants evaluated the statements from 1 (strongly disagree) to 7 (strongly agree).

**Table A5:** *Pre‐assessment*: Entrepreneurial role model—Inspiration/Modeling (modified from Nauta & Kokaly, 2001)

Domain	Item	Mean	SD	Item total correlation	Alpha if item is deleted
IM_01	There is an entrepreneurial person I am trying to be like in my career pursuits	4.14	1.62	.725	.821
IM_02	There is an entrepreneurial person particularly inspirational to me in my career path	4.16	1.62	.772	.809
IM_03	In the career path I am pursuing, there is an entrepreneurial person I admire	4.26	1.59	.725	.822
IM_04	I have a mentor in my potential entrepreneurial career field	3.36	1.65	.542	.867
IM_05	I know of an entrepreneurial person who has a career I would like to pursue	4.15	1.68	.647	.841

*Note*. Participants evaluated the statements from 1 (strongly disagree) to 7 (strongly agree).

**Table A6:** *Pre‐assessment*: Perceived entrepreneurial desirability (Modified from Peterman & Kennedy, 2003)

Domain	Item	Mean	SD	Item total correlation	Alpha if item is deleted
D_1	I would love to start my own business	4.39	1.75	.814	.799
D_2	I would be very tense to start my own business	4.40	1.55	.701	.896
D_3	I would be very enthusiastic to start my own business	4.50	1.66	.814	.798

*Note*. Participants evaluated the statements from 1 (strongly disagree) to 7 (strongly agree).

**Table A7:** *Pre‐assessment*: Perceived entrepreneurial feasibility (Modified from Peterman & Kennedy, 2003)

Domain	Item	Mean	SD	Item total correlation	Alpha if item is deleted
F_1	It will be easy to start my own business	2.81	1.53	.624	.723
F_2	I will be successful when I have my own business	4.31	1.40	.551	.748
F_3	I won't be overworked when I have my own business	3.29	1.60	.541	.750
F_4	I know enough how to start a business	3.08	1.68	.644	.714
F_5	I am sure about myself	4.71	1.62	.452	.780

*Note*. Participants evaluated the statements from 1 (strongly disagree) to 7 (strongly agree).

**Table A8:** *Pre‐assessment*: Perceived entrepreneurial intention (Modified from Liñán & Chen, 2009)

Domain	Item	Mean	SD	Item total correlation	Alpha if item is deleted
I_1	I am ready to do anything to be an entrepreneur	3.28	1.65	.770	.941
I_2	My professional goal is to become an entrepreneur	3.66	1.71	.831	.934
I_3	I will make every effort to start and run my own firm	3.64	1.63	.869	.930
I_4	I am determined to create a firm in the future	3.92	1.69	.864	.930
I_5	I have very seriously thought of starting a firm	3.89	1.77	.830	.934
I_6	I have the firm intention to start a firm some day	4.05	1.72	.824	.935

*Note*. Participants evaluated the statements from 1 (strongly disagree) to 7 (strongly agree).

**Table B1:** *Post‐assessment*: Entrepreneurial role model—Inspiration/Modeling (modified from Nauta & Kokaly, 2001)

Domain	Item	Mean	SD	Item total correlation	Alpha if item is deleted
IM_01	There is an entrepreneurial person I am trying to be like in my career pursuits	4.04	1.62	.796	.879
IM_02	There is an entrepreneurial person particularly inspirational to me in my career path	4.19	1.55	.823	.873
IM_03	In the career path I am pursuing, there is an entrepreneurial person I admire	4.15	1.51	.807	.877
IM_04	I have a mentor in my potential entrepreneurial career field	3.65	1.57	.648	.910
IM_05	I know of an entrepreneurial person who has a career I would like to pursue	4.16	1.53	.755	.888

*Note*. Participants evaluated the statements from 1 (strongly disagree) to 7 (strongly agree).

**Table B2:** *Post‐assessment*: Perceived entrepreneurial desirability (Modified from Peterman & Kennedy, 2003)

Domain	Item	Mean	SD	Item total correlation	Alpha if item is deleted
D_1	I would love to start my own business	4.21	1.81	.864	.875
D_2	I would be very tense to start my own business	4.16	1.72	.796	.929
D_3	I would be very enthusiastic to start my own business	4.19	1.75	.879	.863

*Note*. Participants evaluated the statements from 1 (strongly disagree) to 7 (strongly agree).

**Table B3:** *Post‐assessment*: Perceived entrepreneurial feasibility (Modified from Peterman & Kennedy, 2003)

Domain	Item	Mean	SD	Item total correlation	Alpha if item is deleted
F_1	It will be easy to start my own business	3.13	1.67	.697	.805
F_2	I will be successful when I have my own business	4.15	1.53	.678	.811
F_3	I won't be overworked when I have my own business	3.35	1.62	.631	.823
F_4	I know enough how to start a business	3.31	1.69	.739	.793
F_5	I am sure about myself	4.53	1.65	.543	.846

*Note*. Participants evaluated the statements from 1 (strongly disagree) to 7 (strongly agree).

**Table B4:** *Post‐assessment*: Perceived entrepreneurial intention (Modified from Liñán & Chen, 2009)

Domain	Item	Mean	SD	Item total correlation	Alpha if item is deleted
I_1	I am ready to do anything to be an entrepreneur	3.47	1.73	.804	.953
I_2	My professional goal is to become an entrepreneur	3.79	1.70	.867	.946
I_3	I will make every effort to start and run my own firm	3.78	1.69	.884	.944
I_4	I am determined to create a firm in the future	3.98	1.71	.899	.942
I_5	I have very seriously thought of starting a firm	4.02	1.72	.854	.947
I_6	I have the firm intention to start a firm some day	4.04	1.69	.850	.948

*Note*. Participants evaluated the statements from 1 (strongly disagree) to 7 (strongly agree).

**Table B5:** *Post‐assessment*: Entrepreneurial role model—Inspiration/Modeling (modified from Nauta & Kokaly, 2001)

Domain	Item	Mean	SD	Item total correlation	Alpha if item is deleted
IM_01	There is an entrepreneurial person I am trying to be like in my career pursuits	4.04	1.62	.796	.879
IM_02	There is an entrepreneurial person particularly inspirational to me in my career path	4.19	1.55	.823	.873
IM_03	In the career path I am pursuing, there is an entrepreneurial person I admire	4.15	1.51	.807	.877
IM_04	I have a mentor in my potential entrepreneurial career field	3.65	1.57	.648	.910
IM_05	I know of an entrepreneurial person who has a career I would like to pursue	4.16	1.53	.755	.888

*Note*. Participants evaluated the statements from 1 (strongly disagree) to 7 (strongly agree).

**Table B6:** *Post‐assessment*: Perceived entrepreneurial desirability (Modified from Peterman & Kennedy, 2003)

Domain	Item	Mean	SD	Item total correlation	Alpha if item is deleted
D_1	I would love to start my own business	4.21	1.81	.864	.875
D_2	I would be very tense to start my own business	4.16	1.72	.796	.929
D_3	I would be very enthusiastic to start my own business	4.19	1.75	.879	.863

*Note*. Participants evaluated the statements from 1 (strongly disagree) to 7 (strongly agree).

**Table B7:** *Post‐assessment*: Perceived entrepreneurial feasibility (Modified from Peterman & Kennedy, 2003)

Domain	Item	Mean	SD	Item total correlation	Alpha if item is deleted
F_1	It will be easy to start my own business	3.13	1.67	.697	.805
F_2	I will be successful when I have my own business	4.15	1.53	.678	.811
F_3	I won't be overworked when I have my own business	3.35	1.62	.631	.823
F_4	I know enough how to start a business	3.31	1.69	.739	.793
F_5	I am sure about myself	4.53	1.65	.543	.846

*Note*. Participants evaluated the statements from 1 (strongly disagree) to 7 (strongly agree).

**Table B8:** *Post‐assessment*: Perceived entrepreneurial intention (Modified from Liñán & Chen, 2009)

Domain	Item	Mean	SD	Item total correlation	Alpha if item is deleted
I_1	I am ready to do anything to be an entrepreneur	3.47	1.73	.804	.953
I_2	My professional goal is to become an entrepreneur	3.79	1.70	.867	.946
I_3	I will make every effort to start and run my own firm	3.78	1.69	.884	.944
I_4	I am determined to create a firm in the future	3.98	1.71	.899	.942
I_5	I have very seriously thought of starting a firm	4.02	1.72	.854	.947
I_6	I have the firm intention to start a firm some day	4.04	1.69	.850	.948

*Note*. Participants evaluated the statements from 1 (strongly disagree) to 7 (strongly agree).
